# Layered Ni(OH)_2_-Co(OH)_2_ films prepared by electrodeposition as charge storage electrodes for hybrid supercapacitors

**DOI:** 10.1038/srep39980

**Published:** 2017-01-04

**Authors:** Tuyen Nguyen, Michel Boudard, M. João Carmezim, M. Fátima Montemor

**Affiliations:** 1 CQE - Centro de Química Estrutural, Instituto Superior Técnico, Universidade de Lisboa, 1049-001 Lisboa, Portugal; 2 LMGP, Univ. Grenoble Alpes, CNRS, F-38000 Grenoble, France; 3 ESTSetúbal, Instituto Politécnico de Setúbal, 1959-007 Setúbal, Portugal

## Abstract

Consecutive layers of Ni(OH)_2_ and Co(OH)_2_ were electrodeposited on stainless steel current collectors for preparing charge storage electrodes of high specific capacity with potential application in hybrid supercapacitors. Different electrodes were prepared consisting on films of Ni(OH)_2_, Co(OH)_2_, Ni_1/2_Co_1/2_(OH)_2_ and layered films of Ni(OH)_2_ on Co(OH)_2_ and Co(OH)_2_ on Ni(OH)_2_ to highlight the advantages of the new architecture. The microscopy studies revealed the formation of nanosheets in the Co(OH)_2_ films and of particles agglomerates in the Ni(OH)_2_ films. Important morphological changes were observed in the double hydroxides films and layered films. Film growth by electrodeposition was governed by instantaneous nucleation mechanism. The new architecture composed of Ni(OH)_2_ on Co(OH)_2_ displayed a redox response characterized by the presence of two peaks in the cyclic voltammograms, arising from redox reactions of the metallic species present in the layered film. These electrodes revealed a specific capacity of 762 C g^−1^ at the specific current of 1 A g^−1^. The hybrid cell using Ni(OH)_2_ on Co(OH)_2_ as positive electrode and carbon nanofoam paper as negative electrode display specific energies of 101.3 W h g^−1^ and 37.8 W h g^−1^ at specific powers of 0.2 W g^−1^ and 2.45 W g^−1^, respectively.

Transition metal hydroxides (TMHs) are attractive materials for the design of charge storage (or battery type) electrodes with potential application in hybrid (or asymmetric) supercapacitors due to its high specific charge storage capacity, or high specific capacitance, resulting from Faradaic redox reactions with hydroxyl anions in alkaline electrolytes^1^,^2^,^3^,^4^. The high specific capacity associated to the redox activity is due to the layered structure of the TMHs, characterized by a large interlayer distance, which favors the diffusion of ions from the electrolyte into the bulk material, leading to additional redox reactive sites besides the surface ones^5^. Thus, the charge storage performance of TMH-based electrodes^6^ is less dependent on their surface area, compared to metal oxides based electrodes, due to the contribution from the bulk redox reactions. Nevertheless, for increased charge storage capacity and enhanced electrochemical response, a rational design of TMH-based electrodes is still needed and is attracting a lot of interest in the research community^6^ and different solutions have been reported. For example, interconnected Co(OH)_2_ nanosheet films were prepared by constant potential electrodeposition^7^ and single crystal Ni(OH)_2_ nanoplatelet array films were deposited by hydrothermal method^8^, displayed good charge storage capacity.

Double TMHs have been reported as attractive solutions for preparing electrodes of increased charge storage capacity for hybrid supercapacitors because they present some advantages compared to the corresponding single TMHs^1^,^9^. The co-existence of different transition metal (TM) ions in the double TMHs, the redox response is further enhanced compared to the corresponding single TMHs, thanks to the combination of multiple redox reactions involving the two TMs^10^,^11^. For example, porous layered Co_1−*x*_Ni_*x*_(OH)_2_ films were prepared by potential sweep electrodeposition^12^ and layered Ni-Ti hydroxide nanosheet films were deposited by two-step hydrothermal method^13^, displayed enhanced charge storage capacity. More recently, Ni^3+^ doped Ni-Ti hydroxide monolayer nanosheets were reported, presenting a very high charge capacity with specific capacitance of 2310 F g^−1^ at 1.5 A g^−1 ^14.

Among the double TMHs that have been studied in literature, Ni-Co hydroxides are considered as the most promising double ones for preparing charge storage materials for hybrid supercapacitor electrodes^9^. The charge storage mechanism of TMHs has been discussed in literature^9^,^15^,^16^,^17^, being similar to the mechanism proposed for their oxide derivatives such as NiCo_2_O_4_, that is based on the synergistic redox reaction involving the changes in oxidation states of Ni^2+^/Ni^3+^ and Co^2+^/Co^3+^ with OH^−^ present in KOH or NaOH electrolytes. The redox response of Ni^2+^/Ni^3+^ in Ni(OH)_2_ or NiO occurs at potentials of approximately 0.4–0.6 V vs SCE^18^. The corresponding response of Co^2+^/Co^3+^ in Co(OH)_2_ or Co_3_O_4_ occurs at potentials of approximately 0–0.2 V vs SCE^19^. Their synergistic redox response occurs at approximately 0.2–0.4 V vs SCE^20^, resulting in enhanced charge storage capacity in the Ni-Co hydroxides.

Most of studies performed on double Ni-Co hydroxides have been focused on its synergistic redox response compared to the single TMHs. Thus, it is expectable that electrodes composed of single Ni(OH)_2_ and single Co(OH)_2_ hydroxides designed in a way that combines the redox response from the single Ni^2+^/Ni^3+^ and Co^2+^/Co^3+^ redox reactions, will display enhanced electrochemical response. Thus, in this study we design novel architectures of two-layered films composed of Ni(OH)_2_ on Co(OH)_2_ and Co(OH)_2_ on Ni(OH)_2_ (hereafter denominated Ni(OH)_2_-Co(OH)_2_ and Co(OH)_2_-Ni(OH)_2_, respectively) via a simple two steps electrodeposition route as high charge storage capacity electrodes. For comparative purposes single layers of Ni(OH)_2_, Co(OH)_2_ and Ni_1/2_Co_1/2_(OH)_2_ were also prepared and studied. An optimized two-layered Ni(OH)_2_-Co(OH)_2_ film used as positive electrode in a hybrid cell employed carbon nanofoam paper (CNFP) as negative electrode displayed high charge storage capacity.

## Results and Discussion

**Figure 1:**
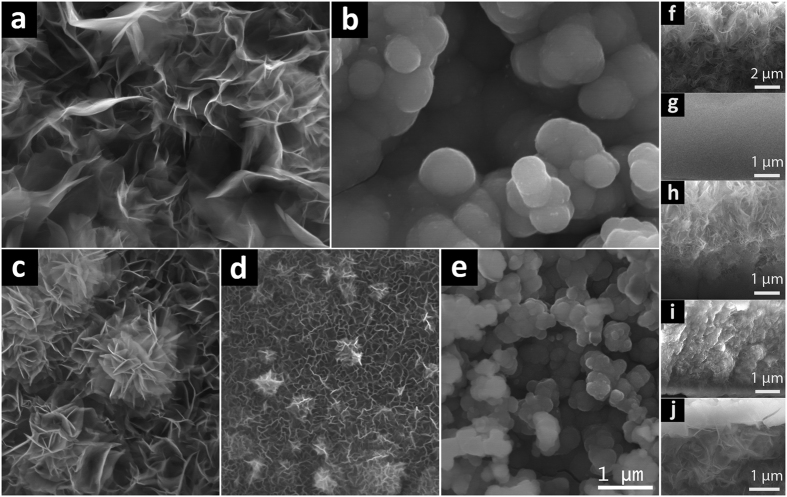
Top-view FEG-SEM images of the (**a**) Co(OH)_2_, (**b**) Ni(OH)_2_, (**c**) Co(OH)_2_-Ni(OH)_2_, (**d**) Ni_1/2_Co_1/2_(OH)_2_ and (**e**) Ni(OH)_2_-Co(OH)_2_ films. The same scale bar in the top-view images is adapted for the other images in the figure. Cross-section FEG-SEM images of the (**f**) Co(OH)_2_, (**g**) Ni(OH)_2_, (**h**) Co(OH)_2_-Ni(OH)_2_, (**i**) Ni_1/2_Co_1/2_(OH)_2_ and (**j**) Ni(OH)_2_-Co(OH)_2_ films.

Figure 1 depicts FEG-SEM images of the surface of the hydroxide films with different architectures electrodeposited on stainless steel substrates. The growth of the surface during electrodeposition involves the reduction of NO_3_^−^ anions and consequent generation of OH^−^ anions and the subsequent reactions of Ni^2+^/Co^2+^ present in the electrolyte with the hydroxyl, leading to the formation of Ni/Co hydroxide films. FEG-SEM images of Co(OH)_2_ and Ni(OH)_2_ films, Fig. 1a and b, show the presence of crumpled nanosheets and agglomerates of nanoparticles. This is in agreement with the surface morphologies of Co_3_O_4_ and NiO films obtained by electrodeposition of their corresponding hydroxides and post thermal transformation (with the preservation of the surface morphology) to form the oxides^21^,^22^. The surface morphology of the Ni_1/2_Co_1/2_(OH)_2_ film, Fig. 1d, reveals the presence of nanosheets, which are almost vertically grown on the substrate and interconnected together, forming a percolation network. This film can probably grow through instantaneous nucleation and subsequent formation of nanosheets^23^. On top of the nanosheets network, dendrites are formed, being randomly distributed over the surface. The surface morphology for Ni_1/2_Co_1/2_(OH)_2_ film revealed different features compared to the corresponding single oxides, probably due to the different preferential growth of single oxides as previously reported^22^.

The surface morphologies of Co(OH)_2_-Ni(OH)_2_ and Ni(OH)_2_-Co(OH)_2_ films, Fig. 1c and e, reveal the presence of a flower-like architecture composed of nanosheets and of agglomerated particles. The nanosheets architecture in Co(OH)_2_-Ni(OH)_2_ is slightly different from that of Co(OH)_2_ films and the Ni(OH)_2_ particle size in the Ni(OH)_2_-Co(OH)_2_ film is smaller compared to the Ni(OH)_2_ film (300 nm vs. 1 μm). This difference can be explained by the fact that Co(OH)_2_ and Ni(OH)_2_ were grown on other hydroxides layers rather than on the stainless steel, resulting in different nucleation process, and thus in distinct surface morphology. It should be noted that, due to low electron conductivity of the bottom layer, the top hydroxide layer favors the grow of a new layer rather than the coverage with nanosheets as reported in previous work for NiCo_2_O_4_/NiCo_2_O_4_ system^24^. It is expected that the morphological features of the layered architecture and the differences in the composition of each electrode, will result in distinct electrochemical response. This issue will be discussed below.

The morphology of the films’ cross-section was studied by FEG-SEM (Fig. 1f–j). The cross-section images of Co(OH)_2_, Ni(OH)_2_ and Ni_1/2_Co_1/2_(OH)_2_ (Fig. 1f,g and i) show the presence of films composed of crumpled nanosheets layer, a dense layer and interconnected nanosheets layer, respectively. This agrees with the top-view FEG-SEM images, indicating the homogeneity of the formed layers. The images of the layered films (Fig. 1h and j) reveal the presence of two layers with different morphologies. Each of them displays morphologies quite similar to that of the single layer films. The Co(OH)_2_ layer is thicker than the Ni(OH)_2_ layer. This can be due to the higher porosity of the Co(OH)_2_ layer compared to the Ni(OH)_2_ layer, leading to an increased volume/area ratio in the Co(OH)_2_ film.

XRD patterns of the Co(OH)_2_, Ni(OH)_2_ and Ni_1/2_Co_1/2_(OH)_2_ films are depicted in Figure S1. The patterns show the typical diffraction of the transition metal hydroxide phases and match with the patterns of the Co(OH)_2_, Ni(OH)_2_ and Ni_1/2_Co_1/2_(OH)_2_ phases reported in literature^7^,^10^,^12^,^25^. The most well-defined peaks, at 2-theta angles of 10.2°, 12.3° and 10.5° for Co(OH)_2_, Ni(OH)_2_ and Ni_1/2_Co_1/2_(OH)_2_ films, are due to the diffraction from the basal planes of layered materials and can be assigned to (001) lattice plane. This angle of Ni_1/2_Co_1/2_(OH)_2_ film is between those of Co(OH)_2_and Ni(OH)_2_, suggesting the formation of the mixed hydroxide film. The broadening of the peaks can be due to the low crystallinity of the films. Thus, XRD results confirm the formation of the hydroxide films.

**Figure 2:**
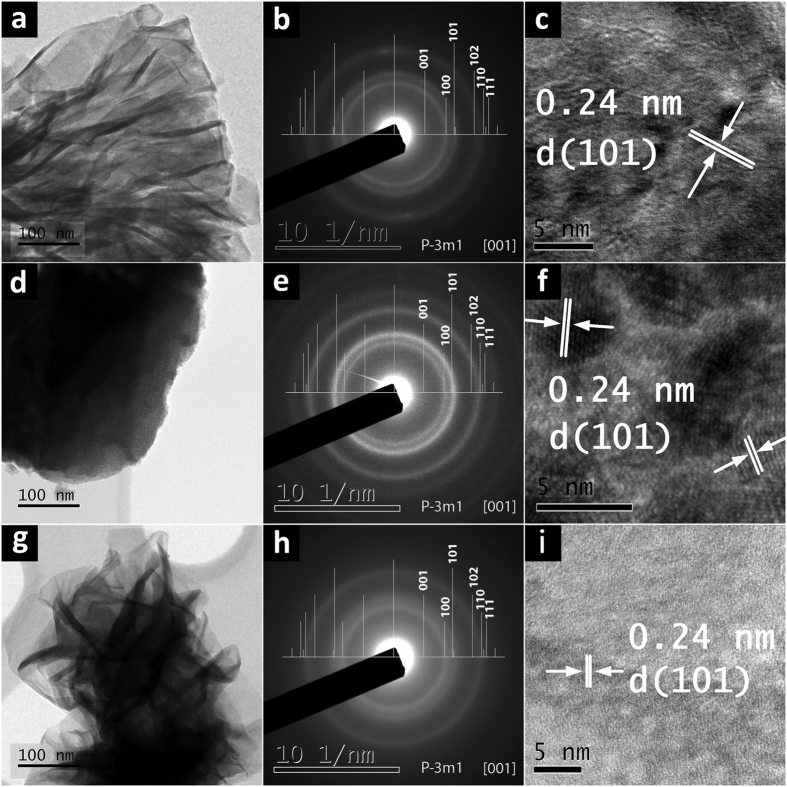
TEM results: low magnified TEM images (left), SAED patterns (middle) and HRTEM images (right) of (**a,b,c**) Co(OH)_2_, (**d,e,f**) Ni(OH)_2_ and (**g,h,i**) Ni_1/2_Co_1/2_(OH)_2_ films.

TEM results the Co(OH)_2_, Ni(OH)_2_ and Ni_1/2_Co_1/2_(OH)_2_ films are depicted in Fig. 2. The Co(OH)_2_ layer and the Ni(OH)_2_ layer in the layered films can be represented by the TEM results obtained for the single layers Co(OH)_2_ and Ni(OH)_2_ films, and thus TEM measurements were not performed on the layered films. Low magnified TEM images reveal the presence of thin and crumpled nanosheets in the Co(OH)_2_ and Ni_1/2_Co_1/2_(OH)_2_ films (Fig. 2a and g) and a dense particle in the Ni(OH)_2_ film (Fig. 2d), in agreement with the FEG-SEM results. The electron diffractions obtained on selected areas (SAED) of these films (Fig. 2b,e and h) show well-defined diffractions rings, revealing the polycrystalline films. The broadening of their rings indicates the formation of nanocrystals or low crystallinity in the films. These rings can be indexed to (100), (101), and (110) lattice planes of the trigonal hydroxide structures (space group P-3m1). Notes that the diffraction from (001) plane is missed, probably due to the oriented growth of hydroxides in [001] direction as reported previously^26^. The d_101_ values are of 2.45 nm, 2.43 nm and 2.44 nm for Co(OH)_2_, Ni(OH)_2_ and Ni_1/2_Co_1/2_(OH)_2_ films, respectively. The d_101_ value of Ni_1/2_Co_1/2_(OH)_2_ films is lower than that of Co(OH)_2_ film and higher than that of Ni(OH)_2_ film, indicating the formation of the mixed hydroxide Ni_1/2_Co_1/2_(OH)_2_. High resolution TEM (HRTEM) results (Fig. 2c,f and i) evidence the lattice fringes, evidencing distances of about 0.24 nm, which are randomly oriented and that correspond to the lattice spacing of (101) planes, thus in agreement with the SAED results. TEM results, which are in agreement with the XRD results, confirm the formation of the hydroxide phases.

**Figure 3:**
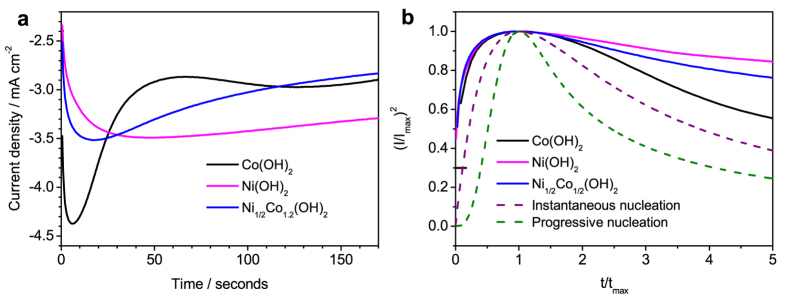
(**a**) Current transients of Co(OH)_2_, Ni(OH)_2_ and Ni_1/2_Co_1/2_(OH)_2_ electrodeposition and (**b**) the corresponding (I/I_max_)^2^ vs. t/t_max_ curves obtained by changing coordinates of these current transients.

Nucleation mechanism of the hydroxide film electrodeposition is studied by examining their current transients (CTs), Fig. 3a. The current response in the CT curves of Co(OH)_2_, Ni(OH)_2_ and Ni_1/2_Co_1/2_(OH)_2_ electrodeposition initially increases and decreases after reaching maxima. The current increase can be due to the increase of nucleus size and number^27^,^28^,^29^. The growth of nuclei occurs under diffusion-controlled process, forming diffusion zone around nuclei^27^,^28^,^29^. The overlap of diffusion zones probably results in the current decrease^27^,^28^,^29^. It is not possible to exact relevant nucleation information from current transients of Ni(OH)_2_ and Co(OH)_2_ electrodeposition on Co(OH)_2_ and Ni(OH)_2_, respectively, because they were deposited on porous hydroxides. The current response reaches the maximum values in the time sequence of Co(OH)_2_, Ni_1/2_Co_1/2_(OH)_2_ and Ni(OH)_2_. This result suggests the nucleation rate is fastest for Co(OH)_2_ electrodeposition and slowest for Ni(OH)_2_ electrodeposition. The Ni_1/2_Co_1/2_(OH)_2_ electrodeposition nucleate at the rate in the middle of Co(OH)_2_ and Ni(OH)_2_ nucleation rates, which is probably a result of the formation of the mixed nuclei.

The current transients are transformed to another coordination, (I/I_max_)^2^ vs. t/t_max_, and compare to instantaneous and progressive nucleation models^29^, Fig. 3b. The experimental curves are close to instantaneous nucleation. Though they deviate from this theoretical curve, previous studies by *in situ* AFM confirmed this behavior occurs under instantaneous nucleation^23^. Thus, the results suggest the hydroxide film electrodeposition instantaneously nucleate.

**Figure 4:**
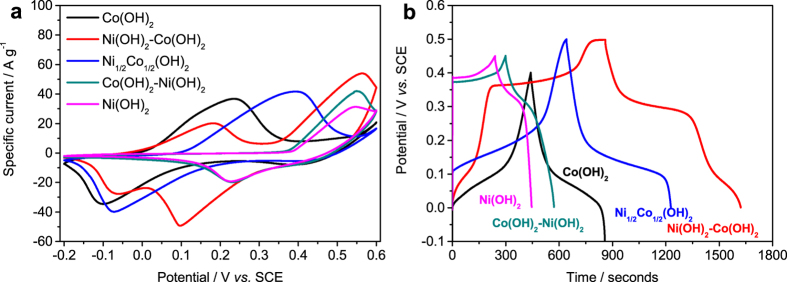
(**a**) Cyclic voltammograms at 20 mV s^−1^ and (**b**) galvanostatic charge-discharge curves at 1 A g^−1^ of Ni(OH)_2_, Co(OH)_2_, Co(OH)_2_-Ni(OH)_2_, Ni_1/2_Co_1/2_(OH)_2_ and Ni(OH)_2_-Co(OH)_2_ films. Potential windows in GCD measurements of the hydroxide films were optimized to exclude to less contribution regime to the charge capacity.

The electrochemical response of the hydroxide films was studied by CV measurements ranging the potential from −0.2 V to 0.6 V with the sweep rate of 20 mV s^−1^ in 1 M KOH electrolytes; the results are shown in Fig. 4a. The CV curves obtained for Co(OH)_2_ and Ni(OH)_2_ show the presence of broad anodic peaks at approximately 0.22 V and 0.54 V, which can be due to the redox reactions involving OH^−^ as below^26^:

For Co(OH)_2_


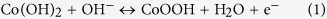



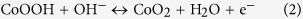


For Ni(OH)_2_


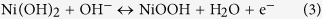


The CV curve of Ni_1/2_Co_1/2_(OH)_2_ also reveals the presence of a broad anodic peak. The redox peak potential is approximately 0.38 V, which is in the middle of the redox peak potentials of its corresponding single oxides: Co(OH)_2_ and Ni(OH)_2_. Furthermore, the current response increases compared to Co(OH)_2_ and Ni(OH)_2_. This result indicates the presence of synergistic redox reactions in the Ni_1/2_Co_1/2_(OH)_2_ film, leading to an increased electrochemical activity.

The CV curves of the Ni(OH)_2_-Co(OH)_2_ films evidence the presence of two peaks, which are approximately at the same potential of the redox response of single Co(OH)_2_ and Ni(OH)_2_. This result evidences that both layers, in the layered film, are contributing to the redox response. The specific current corresponding to the redox reaction of Co species, equation (1 and 2), is lower than that observed for the single Co(OH)_2_ film due to the formation of a thinner Co(OH)_2_ inner layer. The specific current corresponding to the reaction of Ni species, equation (3), increases compared to the values determined for the Ni(OH)_2_ film, probably due to the formation of finer agglomerated particles observed in FEG-SEM image leading to increased redox active sites.

The CV curve of Co(OH)_2_-Ni(OH)_2_ film presents a redox peak in the same potential range observed for the Ni(OH)_2_ films. The specific current at the peaks increases compared to the single Ni(OH)_2_ film. It can also be observed that the onset potential of the anodic peak in the Co(OH)_2_-Ni(OH)_2_ film shifts to more negative potentials compared to the anodic peak in Ni(OH)_2_, evidencing the contribution of the outer Co(OH)_2_ layer. The contribution of the outer Co(OH)_2_ layer, however, is rather small, probably due to the decreased conductivity of Ni(OH)_2_ layer compared to Co(OH)_2_^30^. This point will be further clarified in the next part of the paper.

Overall, considering the separated contribution of the two redox reactions in the layered Ni(OH)_2_-Co(OH)_2_ film, this architecture is expected to display the highest specific capacity values amongst the ones prepared in this work.

To determine the specific capacity values, GCD measurements were performed under a constant current of 1 A g^−1^, Fig. 4b. The specific capacitance values were calculated using equation (7). The potential windows used to measure the specific capacity values of each film were optimized in order to avoid the potential range with minor contribution to the specific capacity in the corresponding films. The results show the presence of two plateaus in the GCD curves of Ni(OH)_2_-Co(OH)_2_ film. The GCD results of the other films revealed only one plateau. This is in a good agreement with the redox response previously observed in the CV curves. The specific capacity values are 417 C g^−1^, 762 C g^−1^, 586 C g^−1^, 272.7 C g^−1^ and 205.6 C g^−1^ at 1 A g^−1^ for Co(OH)_2_, Ni(OH)_2_-Co(OH)_2_, Ni_1/2_Co_1/2_(OH)_2_ and Co(OH)_2_-Ni(OH)_2_ and Ni(OH)_2_ films. The specific capacitance values calculated at 1 A g^−1^ are 834 F g^−1^, 1524 F g^−1^, 1172 F g^−1^, 606 F g^−1^ and 457 F g^−1^ for Co(OH)_2_, Ni(OH)_2_-Co(OH)_2_, Ni_1/2_Co_1/2_(OH)_2_ and Co(OH)_2_-Ni(OH)_2_ and Ni(OH)_2_ films, respectively. The specific capacity of Ni_1/2_Co_1/2_(OH)_2_ increase compared to the single metal hydroxides due to the presence of synergistic redox reactions. The highest specific capacity value determined for the Ni(OH)_2_-Co(OH)_2_ film is due to the separated contribution of the two redox reactions. Thus, this results clearly evidences the advantage of combining multiple redox reaction in the layered Ni(OH)_2_-Co(OH)_2_ film. It is worth to notice that the specific capacity/capacitance values of the Ni(OH)_2_-Co(OH)_2_ film is high, considering that it is deposited on stainless steel which is a flat current collector without any porous channels. Previous publications report that binary Ni-Co hydroxide (NCH) based electrodes, NCH/stainless steel^11^, Ni(OH)_2_-Co(OH)_2_/Ti^31^ formed by co-precipitation and electrodeposited Ni(OH)_2_-Co(OH)_2_/Ti^32^ displayed specific capacitance values of 456 F g^−1^ (205.2 C cm^−2^) at 20 mV s^−1^, 1123 F g^−1^ (561.5 C cm^−2^) at 1 mV s^−1^ and 823 F g^−1^ (411.5 C cm^−2^) at 5 mV s^−1^, respectively. Compared to these values, the new Ni(OH)_2_-Co(OH)_2_ film reported in this work clearly shows high charge storage capacity.

The redox response of the hydroxide films at different rates is evaluated by studying cyclic voltammograms performed at different scanning rates from 5 mV s^−1^ to 50 mV s^−1^, Figure S2a–e. The CVs, in all deposited films, show that the redox peaks are shifted and more separated and that there is an increase of the specific current with the sweep rates. This results reveal the quasi-reversible nature of the redox reactions and suggest the good charge storage at increased rates^33^. The polarization of the electrodes at higher sweep rates results in increased potential difference between the anodic and the cathodic peaks. The shape of the cyclic voltammograms is nearly preserved when increasing sweep rates, indicating easier diffusion of OH^−^ ions into the hydroxide films. This is probably due to characteristics of layered hydroxides, favoring the diffusion of ions^34^. For each film studied, the evolution of the cathodic peak current densities vs. the square root of the sweep rate are showed in Figure S2f. The linear dependence indicates the redox reactions is governed by diffusion-controlled processes^35^.

The high charge storage at increased rate, rate capability, is essential for power application of electrodes working in hybrid supercapacitors. This response is evaluated by analyzing GCD measurements with increased rate up to 10 A g^−1^, Figure S3a–e. The markedly dropped potential and the decreased discharge time, when increasing the specific current, reveal the reduction of the specific capacity. Notably, the response time of Ni(OH)_2_ dropped to zero when applying specific current of 10 A g^−1^. This indicates the intrinsic poor rate capability of Ni(OH)_2_ as reported elsewhere^2^. The specific capacity varies with the applied specific current, Figure S3f. The results show capacity retentions of 81%, 64%, 80%, 25% and 0% when the current increases from 1 A g^−1^ to 10 A g^−1^ for Co(OH)_2_, (b) Ni(OH)_2_-Co(OH)_2_, (c) Ni_1/2_Co_1/2_(OH)_2_, (d) Co(OH)_2_-Ni(OH)_2_ and (e) Ni(OH)_2_, respectively. The reduction of the specific capacity in all the films is probably a result of the less accessible active sites at increased rates. Co(OH)_2_ and Ni_1/2_Co_1/2_(OH)_2_ present the highest rate capability, while Ni(OH)_2_ and Co(OH)_2_-Ni(OH)_2_ present the poorest rate capability. The value of Ni(OH)_2_-Co(OH)_2_ is lower than that of Co(OH)_2_ and Ni_1/2_Co_1/2_(OH)_2_, probably due to the presence of Ni(OH)_2_ layer, which has low rate capability. However, considering the specific capacity values obtained at 10 A g^−1^ of 333.5 C g^−1^, 474.5 C g^−1^ and 487.5 C g^−1^ respectively for Co(OH)_2_ and Ni_1/2_Co_1/2_(OH)_2_ and Ni(OH)_2_-Co(OH)_2_, it is possible to conclude that the two layers films still displayed the highest specific capacity values.

The high charge storage capacity after cycling is also required for supercapacitor electrodes. This test is performed on the Ni(OH)_2_-Co(OH)_2_ electrodes, which achieves the highest specific capacity values, by continuously charge-discharge for 1000 cycles at 10 A g^−1^. The specific capacity, Figure S3g, increases to about 108% after 50 cycles. Then, it slowly decreases to about 71% after 1000 cycles. Even though, the final value is not so high, this value is similar to the values reported for Ni_x_Co_2-x_(OH)_6_/NiCo_2_O_4_ grown on carbon paper^20^. It is worth to note that the continuous charge-discharge test was performed at a high-applied current −10 A g^−1^. A similar test for Ni(OH)_2_/graphite foam presented a capacity retention of 66% after 1000 cycles^36^. The specific capacity values after 1000 cycles is 346 C g^−1^ at 10 A g^−1^, revealing an attractive specific capacity value after cycling.

Both the double layer capacity (*Q*_*v*=∞_, due to the porous morphologies) and the diffusion-controlled redox capacity (due to the redox response) contribute to the total storage capacity of the hydroxide electrodes. A kinetic model describes the dependence of each capacity on potential sweep rate (*v*), performed by cyclic voltammetry, is used to study charge storage mechanism and their capacity contribution to the total capacity of the electrodes^37^. This dependency is described as^37^,^38^,^39^:


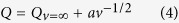



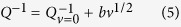


where: *Q* is the total charge at different sweep rates obtained by integrating CV curves, *Q*_*v*=∞_ is the double layer charge and *Q*_*v*=0_ is the maximum total charge can be obtained and *a* and *b* are constants.

**Figure 5:**
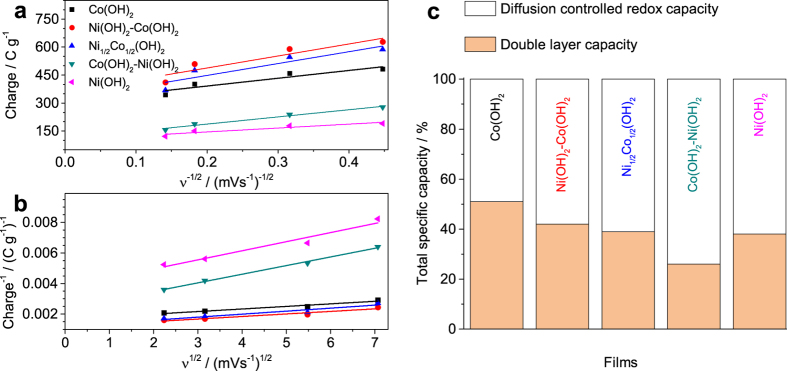
The relation of (**a**) charge (*Q*) versus *v*^−1/2^ and (**b**) of *Q*^−1^ versus *v*^1/2^. (**c**) Contributions of double layer capacity and diffusion-controlled redox capacity to the total capacity of the hydroxide films.

The double layer capacity and the total capacity contributions can be calculated by determining the *y*-axis intercepts in the *Q* versus *v*^−1/2^, and *Q*^−1^ versus *v*^1/2^ plots, Fig. 5a and b. The results, Fig. 5c, represent the contributions of the double layer capacity and the diffusion-controlled redox capacity to the total capacity of the hydroxide films.

As shown in Fig. 5c, for all the films except Co(OH)_2_, the diffusional effects contribution to the total capacity is higher than the double layer ones. This result indicates that the reactions under diffusional control predominantly contribute to the total capacity. The higher contribution of the double layer capacity in the Co(OH)_2_ film can be explained by the formation of the thin crumpled nanosheets morphology that results in increased surface area and increased double layer contribution. This analysis shows that the diffusion-controlled redox contribution is 74% in the Co(OH)_2_-Ni(OH)_2_ film that is higher compared to the Ni(OH)_2_ film −62%. This result shows that the Co(OH)_2_ layer also contributes to the total capacitive response of the electrode. The contribution of the diffusion-controlled redox capacity to the total capacity is similar for the single Ni(OH)_2_-Co(OH)_2_ and Ni_1/2_Co_1/2_(OH)_2_ films, suggesting a marked synergistic effect in the layered film.

To further clarify the difference in redox response of the hydroxide electrodes, current-voltage (I–V) and electrochemical impedance spectroscopy (EIS) measurements were carried out, Figure S4. I-V plots, Figure S4a, show the highest current response in the Co(OH)_2_ film over steel electrode, thus revealing the higher conductivity of Co(OH)_2_ film among the other films. EIS of the hydroxide films, Figure S4b, show no difference at high frequency region. In the medium to low frequency region, the slope of the spectra increases in a sequence of Ni(OH)_2_, Co(OH)_2_-Ni(OH)_2_, Ni_1/2_Co_1/2_(OH)_2_/Ni(OH)_2_-Co(OH)_2_ and Co(OH)_2_ films. This result reveals better capacitive response and lower diffusion resistance of Co(OH)_2_ and Ni(OH)_2_-Co(OH)_2_ films compared to Ni(OH)_2_ and Co(OH)_2_-Ni(OH)_2_ films, respectively. Thus, I-V together with EIS results suggest that the increased conductivity and capacitive response of Co(OH)_2_ layer compared to Ni(OH)_2_ layer result in the improved charge storage performance of Ni(OH)_2_-Co(OH)_2_ films versus Co(OH)_2_-Ni(OH)_2_ films.

The Ni(OH)_2_-Co(OH)_2_ film was used as positive electrode in a two-electrode cell composed of carbon nanofoam paper (CNFP) as negative electrode to evaluate possible application of the new designed electrode in hybrid supercapacitors. This cell is referred as CNFP||Ni(OH)_2_-Co(OH)_2_. CNFP electrode displayed porous structure, Figure S5, and typical double layer capacity in potential ranging from −1.4 to 0 V vs SCE, Figure S6. The charge of the electrodes was balanced using equation (8). This balancing results in the mass difference between the positive electrode and the negative electrode of 1 to 7.1.

**Figure 6:**
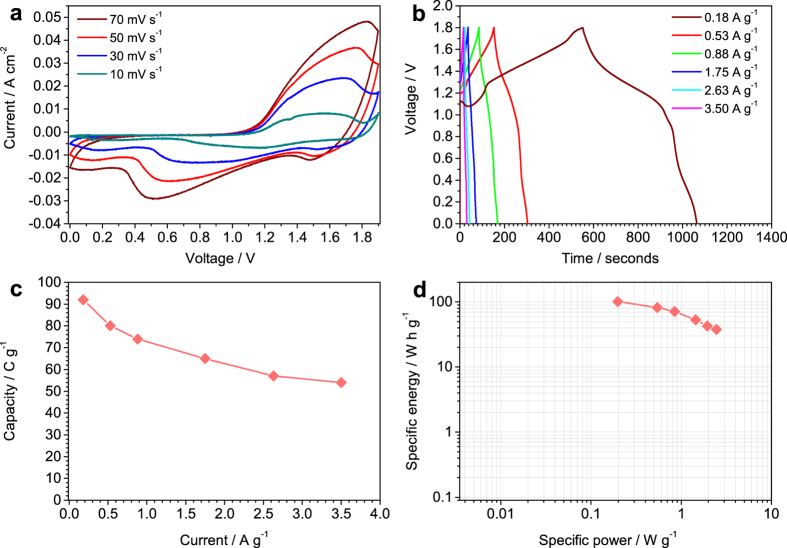
(**a**) Cyclic voltammetry (CV) at different sweep rates, (**b**) galvanostatic charge-discharge curves at different currents, (**c**) capacity versus current values and (**d**) Ragone plot of specific energy versus specific power of the carbon nanofoam paper|| Ni(OH)_2_-Co(OH)_2_ cell.

CV of the cell, Fig. 6a, display redox peaks in the extended working voltage of 1.9 V due to the presence of redox response of Ni(OH)_2_-Co(OH)_2_ electrode and the hybrid cell assembly. The shape of the CV curves at different sweep rates is relatively similar, suggesting a good response rate of the cell. Specific capacity and rate performance of the cell are calculated by performing galvanostatic charge-discharge at different applied currents, Fig. 6b. Specific capacity values are 92 C g^−1^ and 54 C g^−1^ at currents of 0.18 A g^−1^ and 3.5 A g^−1^, Fig. 6c, which retain 58.7% of the specific capacity when the current increases 20 times. Continuous charge-discharge test at the high applied current of 3.5 A g^−1^ shows the capacity retention of 63% after 1000 cycles, Figure S7. The CNFP||Ni(OH)_2_-Co(OH)_2_ hybrid cell display high specific energies of 101.3 W h g^−1^ and 37.8 W h g^−1^ at specific powers of 0.2 W g^−1^ and 2.45 W g^−1^, Fig. 6d, respectively.

Overall, the reported results suggested that new electrodes architectures composed of layered TMHs display enhanced specific capacity values compared to the corresponding double TMH electrodes. Thus, this work opens a novel route for designing TMHs electrodes for enhanced charge storage capacity and increased rate capability.

## Conclusions

In summary, a new electrode architecture composed of layered Ni(OH)_2_ on Co(OH)_2_ was prepared by electrodeposition on stainless steel and studied towards application in hybrid supercapacitors. The electrochemical response of the Ni(OH)_2_ on Co(OH)_2_ electrode compared to Co(OH)_2_, Ni_1/2_Co_1/2_(OH)_2_, Co(OH)_2_-Ni(OH)_2_ and Ni(OH)_2_ revealed enhanced specific capacity response −762 C g^−1^. The high specific capacity was due to the contribution to the redox response of the two layers of hydroxides in the layered film that led to the presence of two redox peaks in the cyclic voltammogram. The contribution of the two hydroxide layers to the total capacity response resulted from an architecture composed of agglomerated particles Ni(OH)_2_ (top layer) over nanosheets Co(OH)_2_ (bottom layer) with high ionic diffusion and better electron conductivity of Co(OH)_2_, allowing the diffusion of hydroxyl ions and redox reactions in both layers of the electrodes. The Ni(OH)_2_ on Co(OH)_2_ architecture displayed good rate capability, with maximum specific capacity values of 487.5 C g^−1^ at 10 A g^−1^ that decreased to 346 C g^−1^ after 1000 of continuous GCD cycles. The carbon nanofoam paper||Ni(OH)_2_-Co(OH)_2_ hybrid cell show high specific energy of 101.3 W h g^−1^ at specific power of 0.2 W g^−1^.

## Methods

### Electrodeposition

Electrodeposition was carried out in potentiostatic mode, in a three-electrode cell, at room temperature, controlled by Radiometer Voltalab PGZ 100 Potentiostat. The cell was composed of stainless steel (AISI 304, Goodfellow) as a substrate (working electrode), platinum foil as counter electrode and the saturated calomel electrode (SCE) as reference electrode. Steel plates used for electrodeposition were previously polished with grit papers, cleaned with deionized water and ethanol and dried in air. The electrolytes used for the deposition were 0.1 M Ni(NO_3_)_2_, 0.1 M Co(NO_3_)_2_ and [0.05 M Ni(NO_3_)_2_ + 0.05 M Co(NO_3_)_2_] for the electrodeposition of Ni(OH)_2_, Co(OH)_2_ and Ni_1/2_Co_1/2_(OH)_2_, respectively. All films were potentiostatically electrodeposited at −1.1 V vs SCE. Single layer films of Ni(OH)_2_, Co(OH)_2_ and Ni_1/2_Co_1/2_(OH)_2_ were deposited with applied charge of −1 C cm^−2^. The Ni:Co ratio in the Ni_1/2_Co_1/2_(OH)_2_ film was similar to the ratio used in the electrolyte (obtained by EDX analysis). Layered films of Ni(OH)_2_-Co(OH)_2_ and Co(OH)_2_-Ni(OH)_2_ were prepared by depositing each layer, separately, following the order in the films, with the applied charge for the deposition of each layer of −0.5 C cm^−2^. The potential values depicted in this work are with reference to the SCE. Loading densities of all the electrodeposited films, weighted by Sartorius micro-balance, were approximately 0.7 mg cm^−2^, thus allowing specific capacity comparison among the electrodes.

### Characterization

The surface morphology (top view and cross section) of the electrodeposited films was studied by field emission gun scanning electron microscopy (FEG-SEM, JEOL 7001 F and FEI QUANTA 250 ESEM microscopes). Cross-section measurements were done by scratching part of the films away from the substrate and observed on 75° tilted sample holder. Elemental analysis was studied by X-ray spectroscopy (EDX) coupled with FEG-SEM.

X-ray diffraction (XRD, Bruker AXS D8 Advance diffractometer) in Bragg-Brentano configuration with Cu K_α_ radiation was used for phase identification.

Transmission electron microscopy (TEM, JEOL 2010 microscope) was used to assess the structural details of the formed film under an acceleration voltage of 200 kV. TEM samples were prepared by scratching the films and collecting the resulting powder by TEM grids to avoid structural transformations that can be induced by other thinning processes.

The electrochemical response and the charge storage ability of the electrodes were studied by cyclic voltammetry (CV), galvanostatic charge-discharge (GCD) and electrochemical impedance spectroscopy (EIS), respectively, in 1 M KOH alkaline electrolytes, using a three-electrode cell as described above.

The specific capacity (*Q*/C g^−1^) and capacitance (*C*/F g^−1^) of the films was calculated from the GCD curves using the formula^40^:


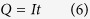



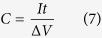


where *I* (A g^−1^), *t* (s) and *∆V* (V) are the GCD specific current, the discharge time and the working potential window, respectively.

Electrochemical impedance spectroscopy (EIS, Gamry FAS2 Femtostat) were performed by applying sine-wave voltage with amplitude of 10 mV at open circuit potential in the frequency range of 10 mHz to 100 kHz.

Current-voltage characteristics of the electrodeposited film over steel electrodes were obtained by linear sweep voltammetry in the voltage range of −0.4 to 0.4 V.

### Two-electrode cell

Two-electrode cell, working in 1 M KOH electrolyte, was assembled using an electrodeposited layered hydroxide film as positive electrode and carbon nanofoam paper (CNFP, Marketech) as negative electrode. Charge balancing was calculated using equation:


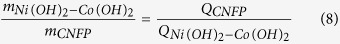


where 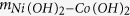 and *m*_*CNFP*_ are loadings of Ni(OH)_2_-Co(OH)_2_ electrode and CNFP electrode; and 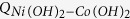 and *Q*_*CNFP*_ are specific charge capacities of Ni(OH)_2_-Co(OH)_2_ electrode and CNFP electrode.

Specific energy (E, W h g^−1^) and specific power (P, W g^−1^) of the cell were calculated from discharge curves using equations:


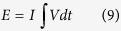



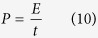


## Additional Information

**How to cite this article**: Nguyen, T. *et al*. Layered Ni(OH)_2_-Co(OH)_2_ films prepared by electrodeposition as charge storage electrodes for hybrid supercapacitors. *Sci. Rep.* **7**, 39980; doi: 10.1038/srep39980 (2017).

**Publisher's note:** Springer Nature remains neutral with regard to jurisdictional claims in published maps and institutional affiliations.

## Supplementary Material

Supplementary Information

## References

[b1] Cheng J. P., Zhang J. & Liu F. Recent development of metal hydroxides as electrode material of electrochemical capacitors. RSC Adv. 4, 38893–38917 (2014).

[b2] Yang G.-W., Xu C.-L. & Li H.-L. Electrodeposited nickel hydroxide on nickel foam with ultrahigh capacitance. Chemical Communications 6537–6539 (2008). 19057771 10.1039/b815647f

[b3] Zhou W.-j., Zhang J., Xue T., Zhao D.-d. & Li H.-l. Electrodeposition of ordered mesoporous cobalt hydroxide film from lyotropic liquid crystal media for electrochemical capacitors. Journal of Materials Chemistry 18, 905–910 (2008).

[b4] Montemor M. F. . In Handbook of Nanoelectrochemistry. (eds. Aliofkhazraei M. & Makhlouf A. S. H.) 1–27 (Springer International Publishing, 2016).

[b5] Wu M.-S. & Huang K.-C. Fabrication of nickel hydroxide electrodes with open-ended hexagonal nanotube arrays for high capacitance supercapacitors. Chemical Communications 47, 12122–12124 (2011). 21998822 10.1039/c1cc14999g

[b6] Yang Q. . Metal Oxide and Hydroxide Nanoarrays: Hydrothermal Synthesis and Applications as Supercapacitors and Nanocatalysts. Progress in Natural Science: Materials International 23, 351–366 (2013).

[b7] Gupta V., Kusahara T., Toyama H., Gupta S. & Miura N. Potentiostatically Deposited Nanostructured α-Co(OH)_2_: A High Performance Electrode Material for Redox-Capacitors. Electrochemistry Communications 9, 2315–2319 (2007).

[b8] Li J., Zhao W., Huang F., Manivannan A. & Wu N. Single-Crystalline Ni(OH)_2_ and NiO Nanoplatelet Arrays as Supercapacitor Electrodes. Nanoscale 3, 5103–5109 (2011).

[b9] Chen H., Hu L., Chen M., Yan Y. & Wu L. Nickel–Cobalt Layered Double Hydroxide Nanosheets for High-performance Supercapacitor Electrode Materials. Advanced Functional Materials 24, 934–942 (2014).

[b10] Gupta V., Gupta S. & Miura N. Potentiostatically Deposited Nanostructured Co_x_Ni_1−x_ Layered Double Hydroxides as Electrode Materials for Redox-Supercapacitors. Journal of Power Sources 175, 680–685 (2008).

[b11] Salunkhe R. R., Jang K., Lee S.-w. & Ahn H. Aligned nickel-cobalt hydroxide nanorod arrays for electrochemical pseudocapacitor applications. RSC Adv. 2, 3190–3193 (2012).

[b12] Kulkarni S. B. . Potentiodynamic Deposition of Composition Influenced Co_1−x_Ni_x_ LDHs Thin Film Electrode for Redox Supercapacitors. International Journal of Hydrogen Energy 38, 4046–4053 (2013).

[b13] Gu Y. . NiTi Layered Double Hydroxide Thin Films for Advanced Pseudocapacitor Electrodes. J. Mater. Chem. A 1, 10655–10661 (2013).

[b14] Zhao Y. . Ni^3+^ doped monolayer layered double hydroxide nanosheets as efficient electrodes for supercapacitors. Nanoscale 7, 7168–7173 (2015). 25815801 10.1039/c5nr01320h

[b15] Xie L. . Co_x_Ni_1−x_ double hydroxide nanoparticles with ultrahigh specific capacitances as supercapacitor electrode materials. Electrochimica Acta 78, 205–211 (2012).

[b16] Wang X., Sumboja A., Lin M., Yan J. & Lee P. S. Enhancing electrochemical reaction sites in nickel–cobalt layered double hydroxides on zinc tin oxide nanowires: a hybrid material for an asymmetric supercapacitor device. Nanoscale 4, 7266–7272 (2012). 23076678 10.1039/c2nr31590d

[b17] Tao Y. . Nickel–cobalt double hydroxides microspheres with hollow interior and hedgehog-like exterior structures for supercapacitors. Journal of Materials Chemistry 22, 23587–23592 (2012).

[b18] Hu C.-C., Chen J.-C. & Chang K.-H. Cathodic deposition of Ni(OH)_2_ and Co(OH)_2_ for asymmetric supercapacitors: Importance of the electrochemical reversibility of redox couples. Journal of Power Sources 221, 128–133 (2013).

[b19] Zhao C. . Ultrahigh capacitive performance from both Co(OH)_2_/graphene electrode and K_3_Fe(CN)_6_ electrolyte. Sci. Rep. 3 (2013). 10.1038/srep02986 PMC3798881 24136136

[b20] Huang L. . Nickel–Cobalt Hydroxide Nanosheets Coated on NiCo_2_O_4_ Nanowires Grown on Carbon Fiber Paper for High-Performance Pseudocapacitors. Nano Lett. 13, 3135–3139 (2013). 23755979 10.1021/nl401086t

[b21] Yuan C. . Growth of ultrathin mesoporous Co_3_O_4_ nanosheet arrays on Ni foam for high-performance electrochemical capacitors. Energy & Environmental Science 5, 7883–7887 (2012).

[b22] Nguyen T., Boudard M., Rapenne L., Carmezim M. J. & Montemor M. F. Morphological changes and electrochemical response of mixed nickel manganese oxides as charge storage electrodes. Journal of Materials Chemistry A 3, 10875–10882 (2015).

[b23] Nguyen T., Carmezim M. J. & Montemor M. F. Current transient and *in-situ* AFM studies of initial growth stages of electrochemically deposited nickel cobalt hydroxide nanosheet films. Physical Chemistry Chemical Physics 18, 12368–12374 (2016). 27087173 10.1039/c6cp00709k

[b24] Liu X. . Hierarchical NiCo_2_O_4_@NiCo_2_O_4_ Core/Shell Nanoflake Arrays as High-Performance Supercapacitor Materials. ACS Appl. Mater. Interfaces 5, 8790–8795 (2013). 23937272 10.1021/am402681m

[b25] Jing M. . Alternating Voltage Introduced NiCo Double Hydroxide Layered Nanoflakes for an Asymmetric Supercapacitor. ACS Appl. Mater. Interfaces 7, 22741–22744 (2015). 26435064 10.1021/acsami.5b05660

[b26] Nguyen T. . Structural evolution, magnetic properties and electrochemical response of MnCo_2_O_4_ nanosheet films. RSC Adv. 5, 27844–27852 (2015).

[b27] Gunawardena G. A., Hills G. J. & Montenegro I. Potentiostatic studies of electrochemical nucleation. Electrochimica Acta 23, 693–697 (1978).

[b28] Hills G. J., Schiffrin D. J. & Thompson J. Electrochemical nucleation from molten salts—I. Diffusion controlled electrodeposition of silver from alkali molten nitrates. Electrochimica Acta 19, 657–670 (1974).

[b29] Scharifker B. & Hills G. Theoretical and experimental studies of multiple nucleation. Electrochimica Acta 28, 879–889 (1983).

[b30] Nogi K., Naito M. & Yokoyama T. Nanoparticle technology handbook. (Elsevier, 2012).

[b31] Han J., Roh K. C., Jo M. R. & Kang Y.-M. A novel co-precipitation method for one-pot fabrication of a Co-Ni multiphase composite electrode and its application in high energy-density pseudocapacitors. Chemical Communications 49, 7067–7069 (2013). 23817341 10.1039/c3cc43350a

[b32] Zhong J.-H. . Co_3_O_4_/Ni(OH)_2_ composite mesoporous nanosheet networks as a promising electrode for supercapacitor applications. Journal of Materials Chemistry 22, 5656–5665 (2012).

[b33] Gujar T. P. . Spray deposited amorphous RuO_2_ for an effective use in electrochemical supercapacitor. Electrochemistry Communications 9, 504–510 (2007).

[b34] Guo X. L. . Nickel-Manganese Layered Double Hydroxide Nanosheets Supported on Nickel Foam for High-performance Supercapacitor Electrode Materials. Electrochimica Acta 194, 179–186 (2016).

[b35] Bard A. & Faulkner L. Electrochemical Methods: Fundamentals and Applications. (John Wiley & Sons, Inc, 2001).

[b36] Ji J. . Nanoporous Ni(OH)_2_ Thin Film on 3D Ultrathin-Graphite Foam for Asymmetric Supercapacitor. ACS Nano 7, 6237–6243 (2013). 23758135 10.1021/nn4021955

[b37] Ardizzone S., Fregonara G. & Trasatti S. “Inner” and “outer” active surface of RuO_2_ electrodes. Electrochimica Acta 35, 263–267 (1990).

[b38] Nguyen T. . Hybrid nickel manganese oxide nanosheet-3D metallic dendrite percolation network electrodes for high-rate electrochemical energy storage. Nanoscale 7, 12452–12459 (2015). 26135715 10.1039/c5nr02888d

[b39] Nguyen T., Boudard M., Carmezim M. J. & Montemor M. F. Hydrogen bubbling-induced micro/nano porous MnO_2_ films prepared by electrodeposition for pseudocapacitor electrodes. Electrochimica Acta 202, 166–174 (2016).

[b40] Nguyen T., João Carmezim M., Boudard M. & Fátima Montemor M. Cathodic electrodeposition and electrochemical response of manganese oxide pseudocapacitor electrodes. International Journal of Hydrogen Energy 40, 16355–16364 (2015).

