# Supplementary material for: Layered Ni(OH)2-Co(OH)2 films prepared by electrodeposition as charge storage electrodes for hybrid supercapacitors
Source: Sci Rep. 2017 Jan 4;7:39980. doi: 10.1038/srep39980 (PMC5209710; doi:10.1038/srep39980)
Supplement: Supplementary Information [file srep39980-s1.pdf]

Layered $Ni(OH)_2$-$Co(OH)_2$ films prepared by electrodeposition as charge storage electrodes for hybrid supercapacitors

Tuyen Nguyen[*,a,b], Michel Boudard[b], M. João Carmezim[a,c], M. Fátima Montemor[a]

[a] CQE - Centro de Química Estrutural, Instituto Superior Técnico, Universidade de Lisboa, 1049-001 Lisboa, Portugal. [*]Email: nguyen.tuyen@tecnico.ulisboa.pt

[b] LMGP, Univ. Grenoble Alpes, CNRS, F-38000 Grenoble, France.

[c] ESTSetúbal, Instituto Politécnico de Setúbal, 1959-007 Setúbal, Portugal.

Supporting Information

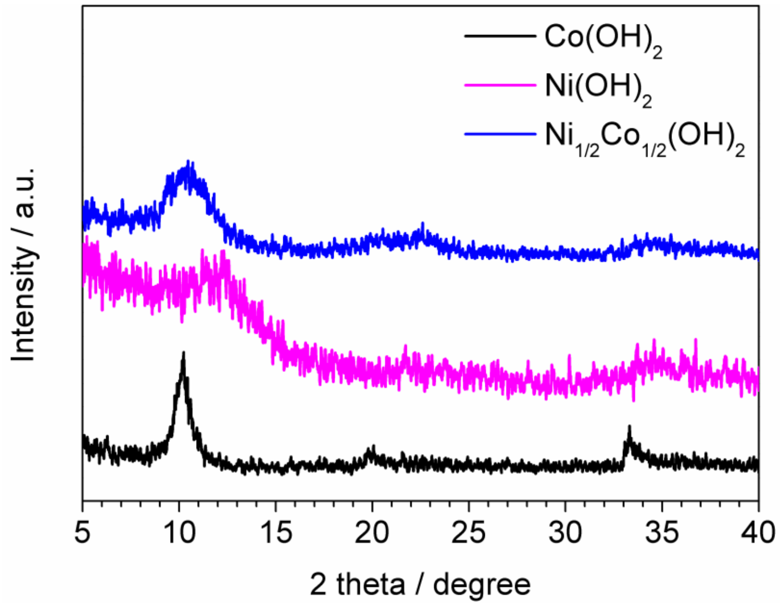

Figure S1. XRD patterns of the (a) $Co(OH)_2$, (b) $Ni(OH)_2$ and (c) $Ni_{1/2}Co_{1/2}(OH)_2$ films.

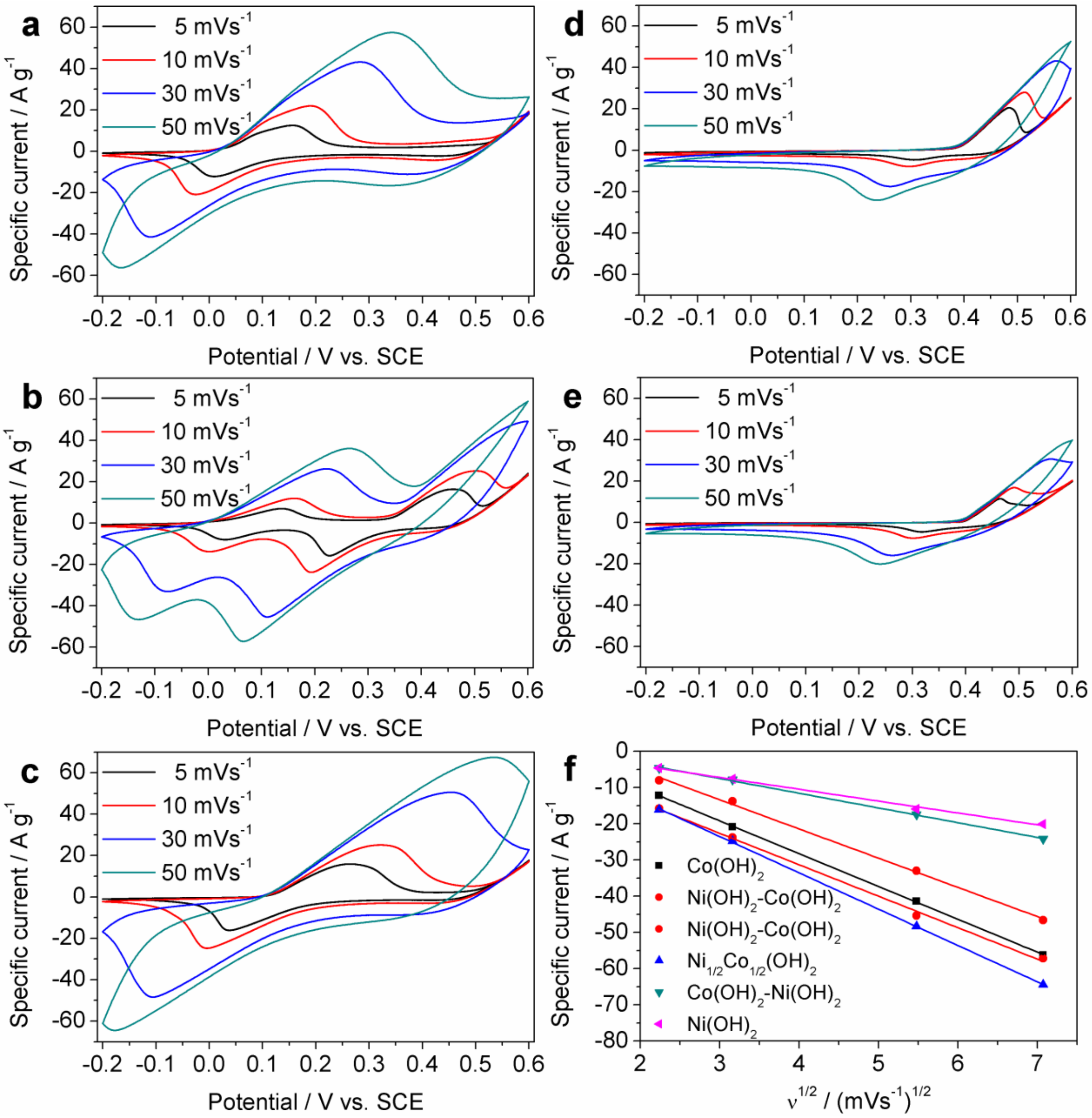

Figure S2. Cyclic voltammograms at different sweep rates of 5 mV s^{-1}, 10 mV s^{-1}, 30 mV s^{-1} and 50 mV s^{-1} of (a) $Co(OH)_2$, (b) $Ni(OH)_2$-$Co(OH)_2$, (c) $Ni_{1/2}Co_{1/2}(OH)_2$, (d) $Co(OH)_2$-$Ni(OH)_2$ and (e) $Ni(OH)_2$. (f) Relation of specific current peaks with the square root of sweep rates.

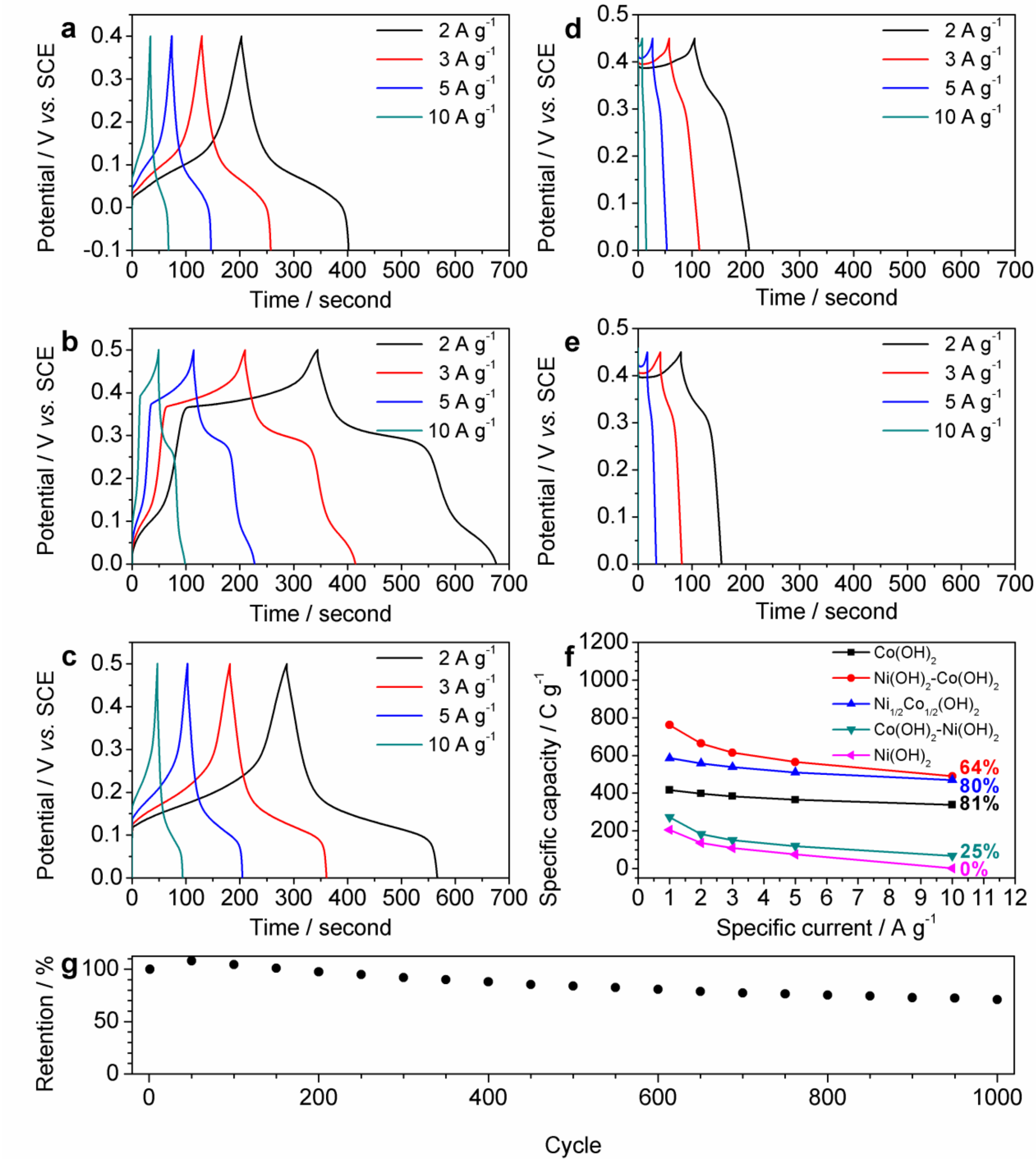

Figure S3. Charge-discharge at different constant currents of 2 A g^{-1}, 3 A g^{-1}, 5 A g^{-1} and 10 A g^{-1} of (a) $Co(OH)_2$, (b) $Ni(OH)_2$-$Co(OH)_2$, (c) $Ni_{1/2}Co_{1/2}(OH)_2$, (d) $Co(OH)_2$-$Ni(OH)_2$ and (e) $Ni(OH)_2$. (f) The relation of specific capacity values with increasing specific current of the prepared films. (g) Continuous charge-discharge cycling of the $Ni(OH)_2$-$Co(OH)_2$ film at 10 A g^{-1} for 1000 cycles.

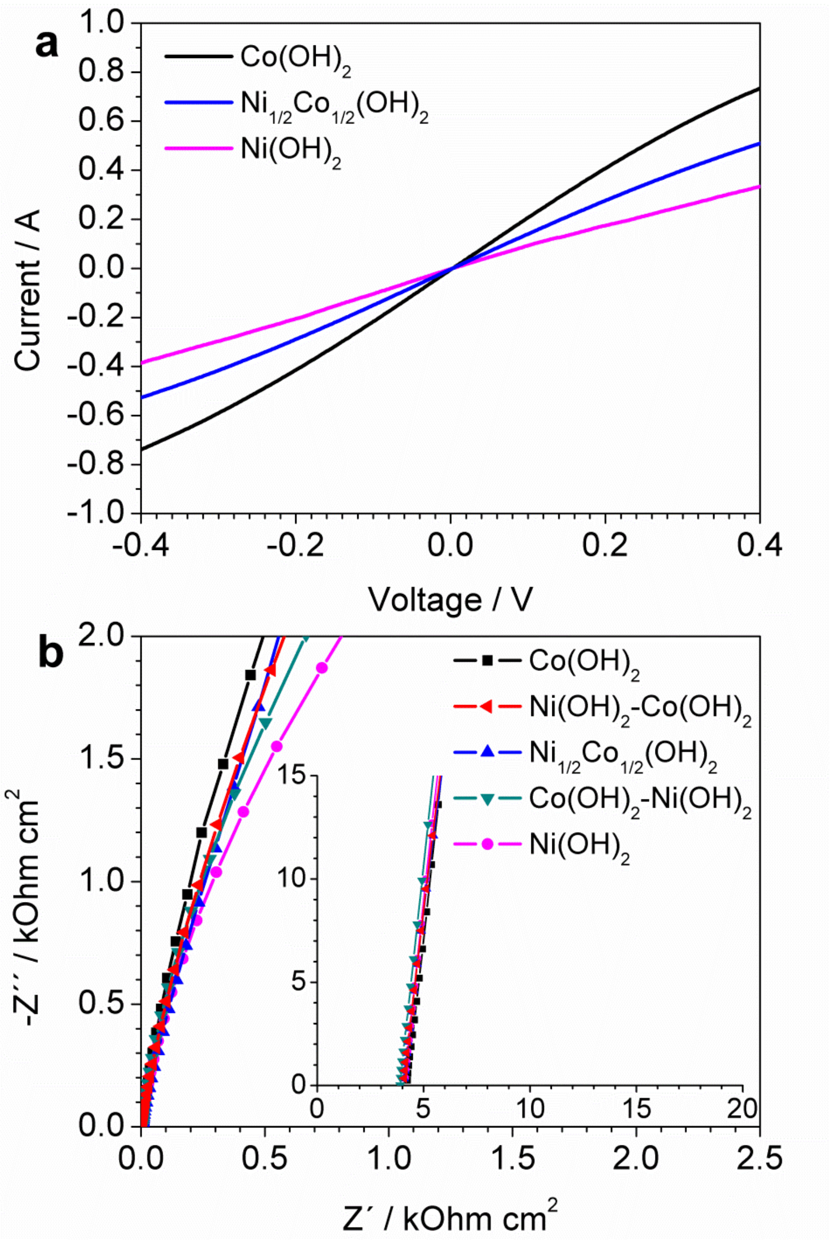

Figure S4. (a) Current-voltage (I-V) curves and (b) electrochemical impedance spectra (EIS, Nyquist plot) of the hydroxide electrodes. Inset is enlarged EIS spectra at high frequency region.

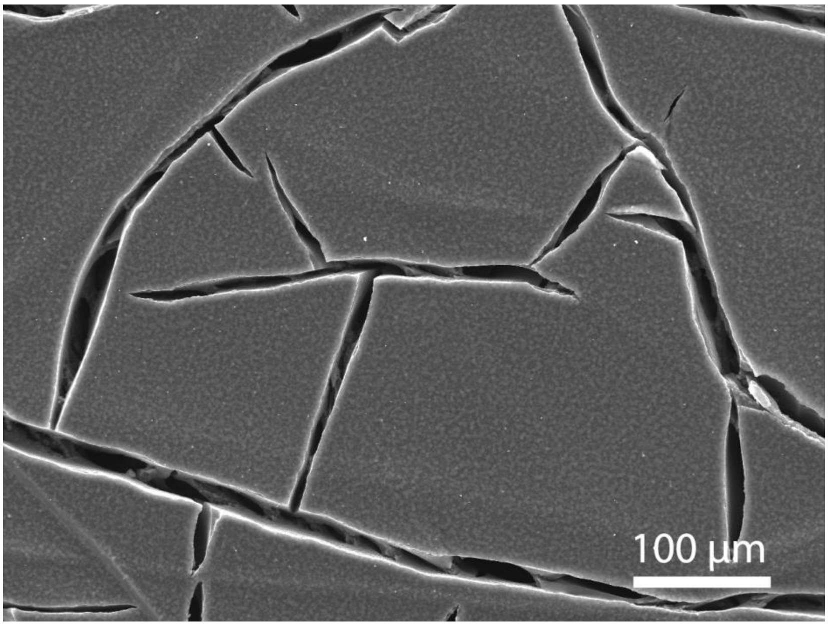

Figure S5. FEG-SEM image of carbon nanofoam paper (CNFP) electrode.

Carbon nanofoam paper (CNFP, Marketech) display porous structure with specific surface area (BET, Brunauer-Emmett-Teller) of 400 m^2 g^{-1} and average pore size of 0.7 nm [1].

[1] Carbon Nanofoam, http://mkt-intl.com/materials/aerogel/carbon-nanofoam/.

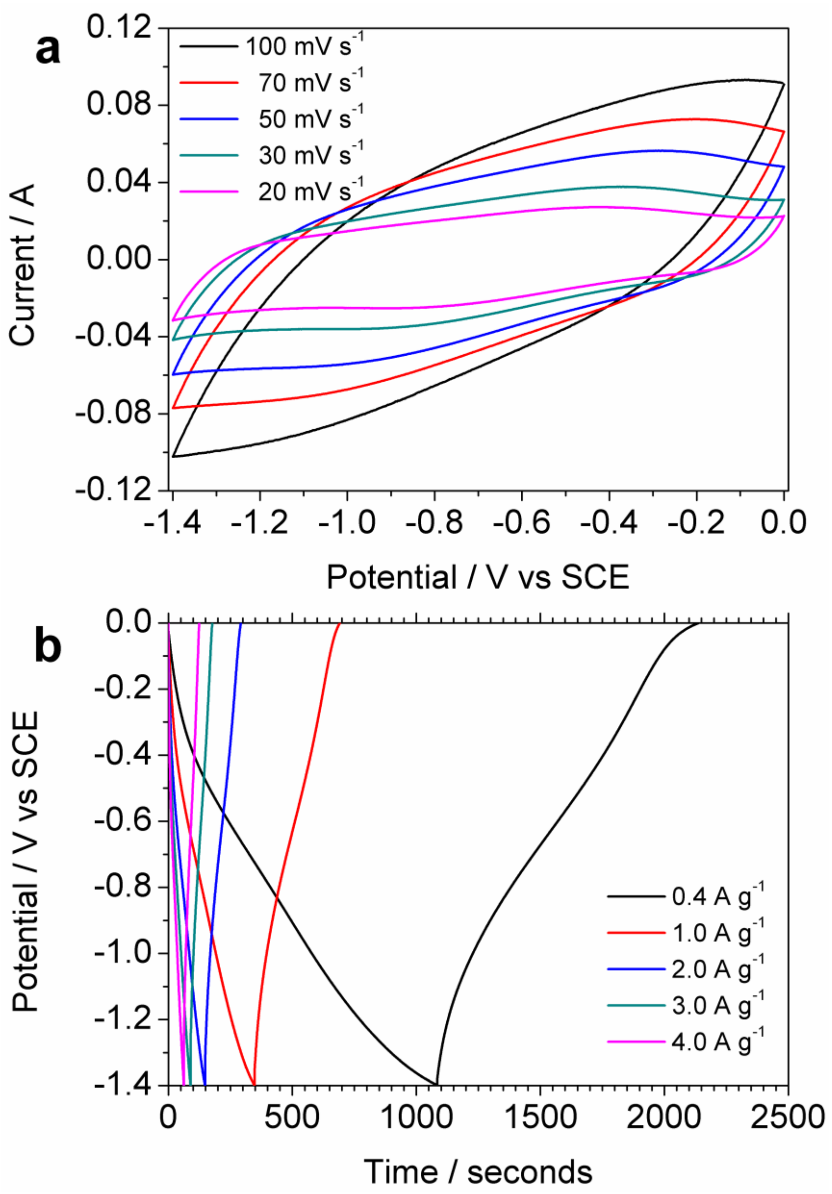

Figure S6. (a) Cyclic voltammograms at different sweep rates and (b) charge-discharge curves at different currents of carbon nanofoam paper electrode in 1 M KOH in a potential window ranging from -1.4 to 0 V vs. SCE.

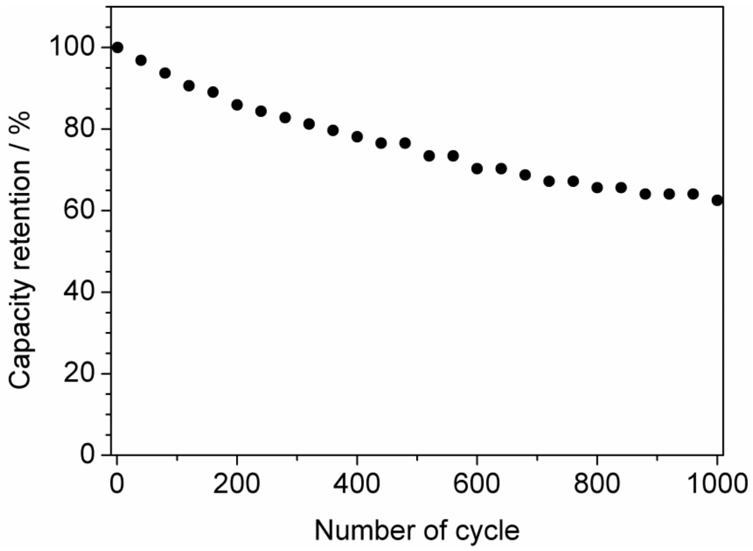

Figure S7. Capacity retention of the carbon nanofoam paper|| $Ni(OH)_2$-$Co(OH)_2$ hybrid cell by continuous charge-discharge at 3.5 A g^{-1} for 1000 cycles.
